# Supplementary material for: ZDHHC20-mediated S-palmitoylation of YTHDF3 stabilizes MYC mRNA to promote pancreatic cancer progression
Source: Nat Commun. 2024 May 31;15:4642. doi: 10.1038/s41467-024-49105-3 (PMC11143236; doi:10.1038/s41467-024-49105-3)
Supplement: Supplementary file 1 — Supplementary Information [file 41467_2024_49105_MOESM1_ESM.pdf]

Supplemental information

ZDHHC20-Mediated S-Palmitoylation of YTHDF3 Stabilizes MYC mRNA to Promote Pancreatic Cancer Progression

Authors:
Huan Zhang[1], Yan Sun[2], Zhaokai Wang[1], Xiaoju Huang[3], Lu Tang[4 *], Ke Jiang[1 *], Xin Jin[5,6 *]

Institutes:
[1]Department of Thoracic Surgery, Union Hospital, Tongji Medical College, Huazhong University of Science and Technology, Wuhan, 430022, China.
[2]Department of Pancreatic Surgery, Union Hospital, Tongji Medical College, Huazhong University of Science and Technology, Wuhan 430022, China
[3]Cancer center, Union Hospital, Tongji Medical College, Huazhong University of Science and Technology, Wuhan 430022, China.
[4]Institute of Hematology, Union Hospital, Tongji Medical College, Huazhong University of Science and Technology, Wuhan, 430022, China.
[5]Department of Urology, the Second Xiangya Hospital, Central South University, Changsha, Hunan 410011, China
[6]Uro-Oncology Institute of Central South University, Changsha, Hunan 410011, China

The file includes:

- Supplementary figures and figure legends
- Supplementary Table S1. Sequence of primers and gene specific shRNAs, sgRNAs & siRNAs
- Supplementary Table S2. The sequences of primers, oligos and m6A-oligos used in this study

Supplementary figures and figure legends

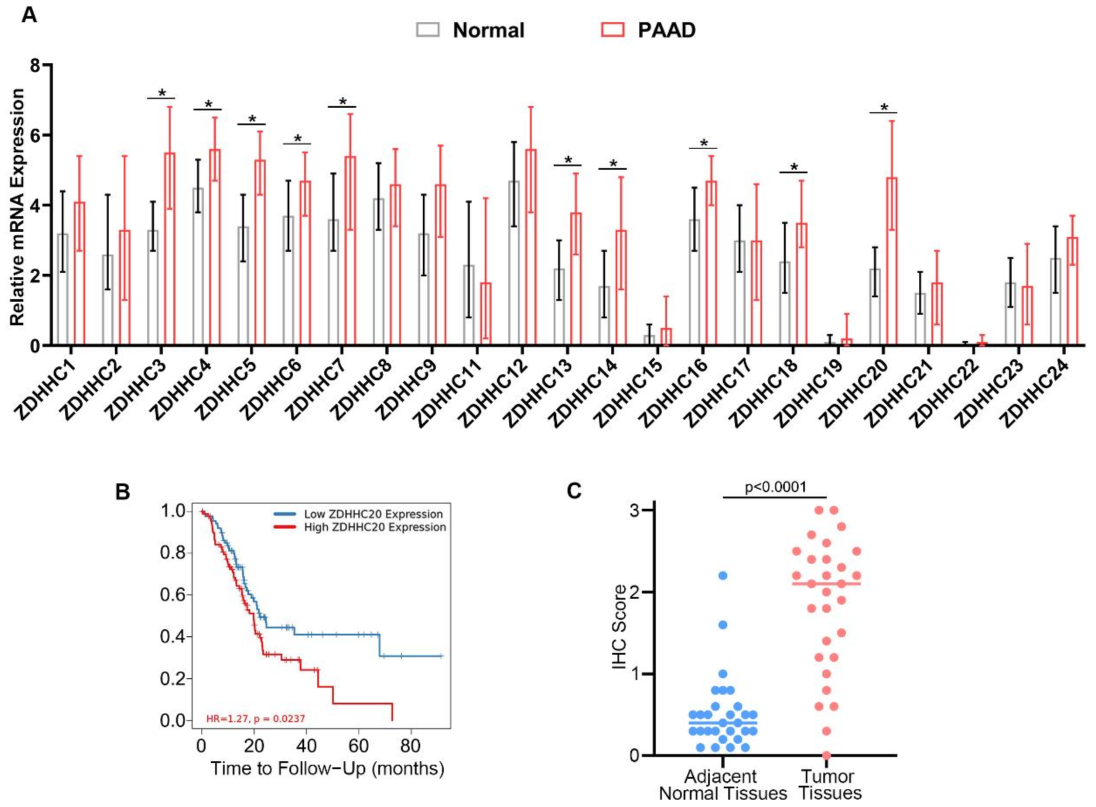

Figure S1. Aberrant ZDHHC20 upregulation predicts unfavorable prognosis in pancreatic cancer

(A) The expression of all known palmitoyl acyltransferases in pancreatic cancer based on the Gene Expression Profiling Interactive Analysis (GEPIA) web server; T: Tumor, n=179; N: Normal n=171, statistical data are presented as median with 95% confidence interval, two-tailed Wilcoxon signed rank test.

(B) Survival analysis of ZDHHC20 in PDAC based on data from the Tumor Immune Estimation Resource (TIMER) database, High: n=79; Low: n=79, the log-rank (Mantel-Cox) test for survival analysis.

(C) Dot plots of IHC analysis for ZDHHC20 on TMA containing a cohort of pancreatic cancer samples (n AN and PDAC =29, two-tailed unpaired *t* test). Source data are provided as a Source Data file.

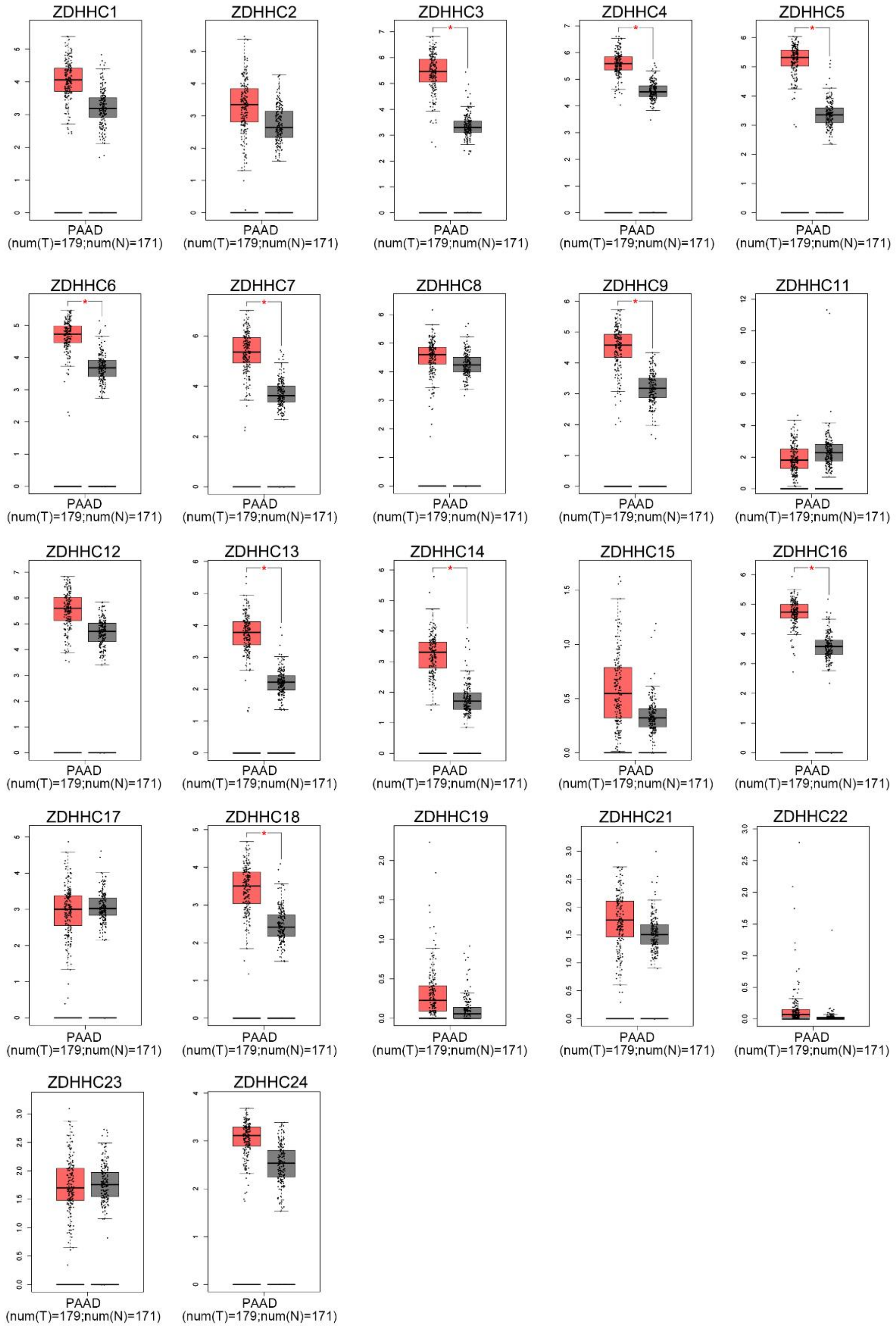

Figure S2. The expression of all ZDHHCs in pancreatic cancer

Boxplot of the expression of all known palmitoyl acyltransferases in pancreatic cancer based on the GEPIA web server, two-tailed Wilcoxon signed rank test. T: Tumor; N: Normal; $P < 0.01$*. The box plots are defined in terms of median, upper quartile, lower quartile and 95% confidence interval.

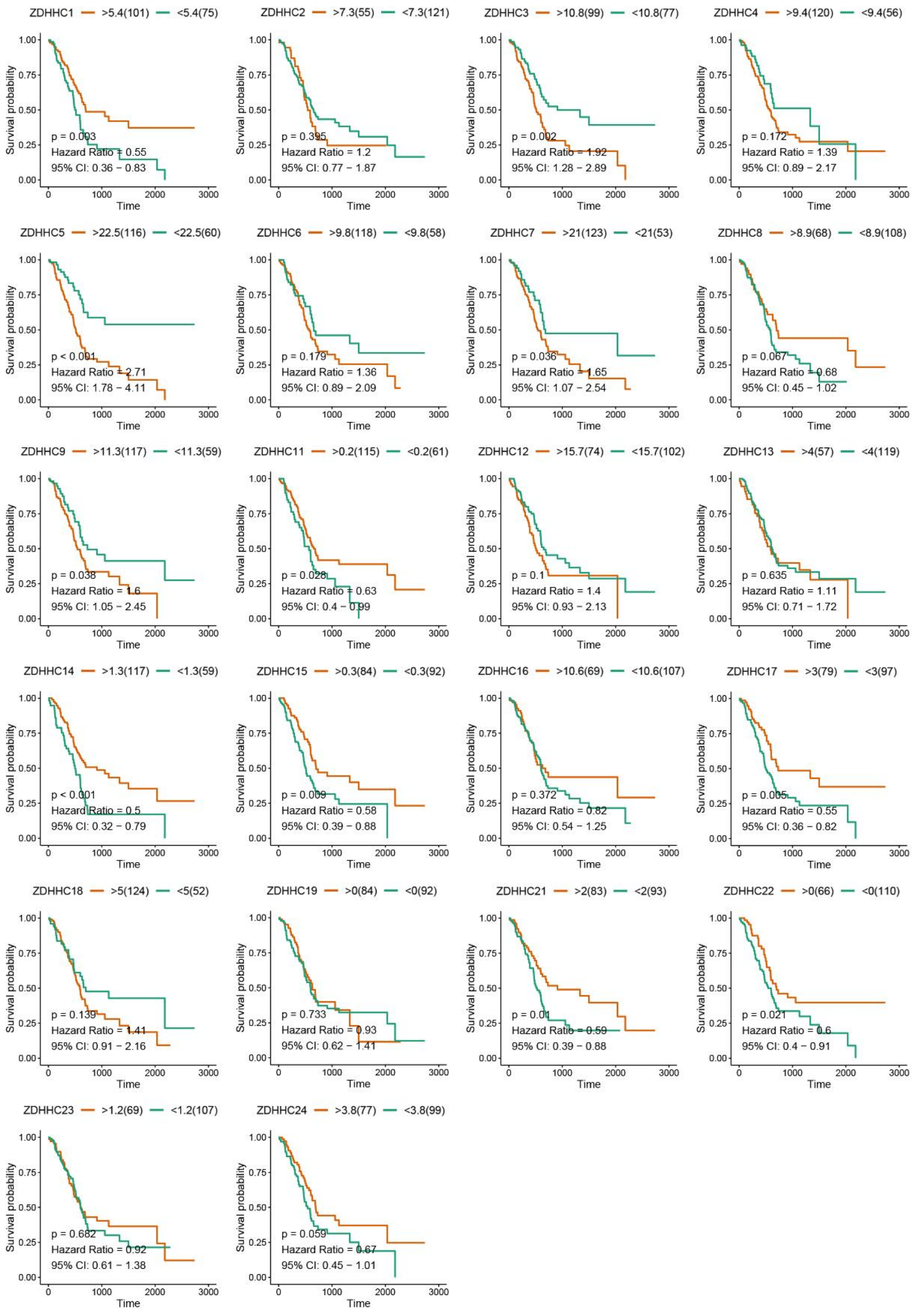

Figure S3. The overall survival of all ZDHHCs in pancreatic cancer

Survival analysis of all known palmitoyl acyltransferases in pancreatic cancer in a pancreatic cancer dataset from TCGA (Orange: ZDHHCs high expression; Green: ZDHHCs low expression, n is in brackets, the log-rank test for survival analysis).

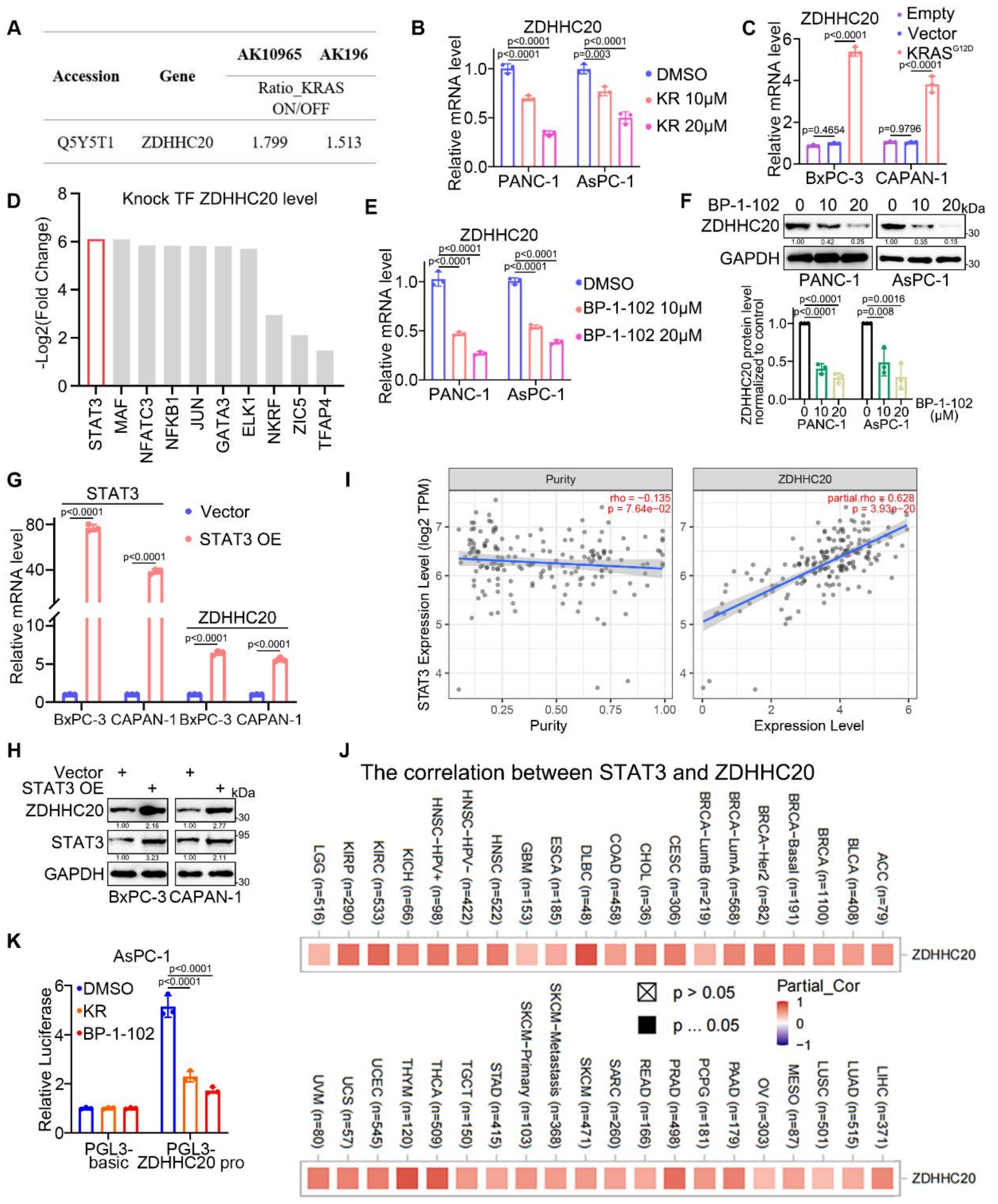

Figure S4. The overall survival of all ZDHHCs in pancreatic cancer

(A) ZDHHC20 is one of the KRAS up-regulated proteins in different pancreatic ductal adenocarcinoma (PDAC) cell lines (AK10965, AK196). (B) RT-qPCR analysis for the mRNA expression level of ZDHHC20 in PANC-1 and AsPC-1 cells treated with KRAS^{G12D} inhibitor (KR: KRpep-2d), n=3 biologically independent experiments, one-way ANOVA. (C) RT-qPCR analysis for the mRNA expression level of ZDHHC20 in BxPC-3 and CAPAN-1 cells infected with KRAS^{G12D} plasmids, n=3 biologically independent experiments, one-way ANOVA. (D) The analysis of the transcription factors that target ZDHHC20 based on the KnockTF platform. (E) RT-qPCR analysis for the mRNA expression level of ZDHHC20 in PANC-1 and

AsPC-1 cells treated with STAT3 inhibitor (BP-1-102), n=3 biologically independent experiments, one-way ANOVA. (F) Western blot analysis for the expression of ZDHHC20 in PANC-1 and AsPC-1 cells treated with STAT3 inhibitor (BP-1-102), n=3 biologically independent experiments, one-way ANOVA. (G) RT-qPCR analysis for the mRNA expression level of ZDHHC20 in BxPC-3 and CAPAN-1 cells infected with STAT3 plasmids, n=3 biologically independent experiments, two-tailed unpaired *t* test. (H) Western blot analysis for the expression of ZDHHC20 in BxPC-3 and CAPAN-1 cells infected with KRASG12D plasmids. (I, J) TIMER2.0 showed purity-adjusted correlation between STAT3 and ZDHHC20 in various cancer types especially including PAAD. (K) The dual-luciferase reporter assay for the promoter region binding affinity of ZDHHC20 in PANC-1 cells treated with KRpep-2d (20μM) or BP-1-102 (20μM), n=3 biologically independent experiments, one-way ANOVA. Statistical data presented in results (B, C, E-G, K) show mean values ± SD. Similar results for (F, H) panels were obtained in three independent experiments. Source data are provided as a Source Data file.

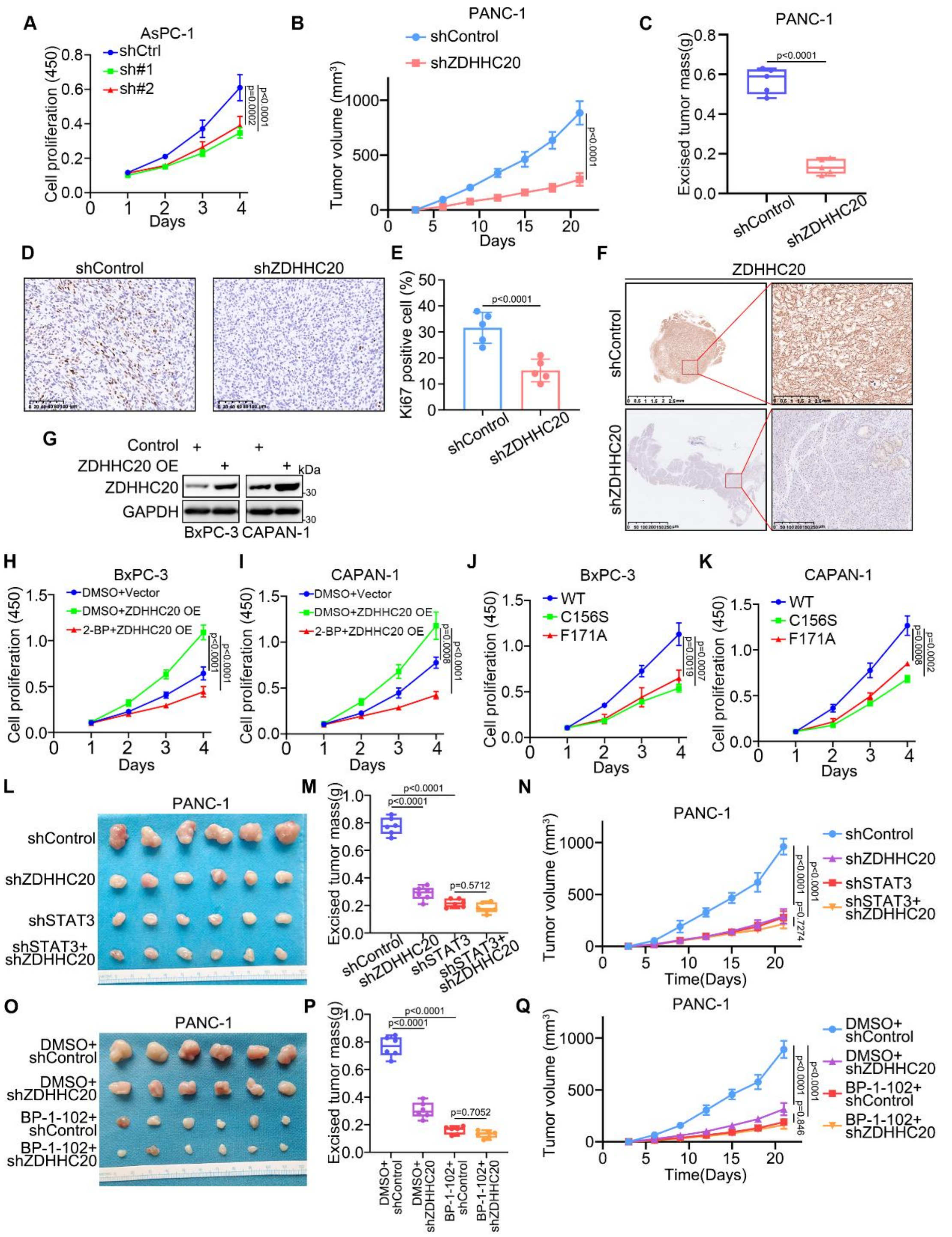

Figure S5. ZDHHC20 promotes pancreatic cancer progression in a palmitoylation-dependent manner

(A) AsPC-1 cells infected with lentivirus vectors expressing control or ZDHHC20 specific shRNAs were harvested for CCK-8 assay, n=3 biologically independent experiments, two-way ANOVA. (B-E) PANC-1 cells infected with lentivirus vectors expressing control or ZDHHC20 specific shRNAs. Cells were injected subcutaneously into the nude mice for xenografts assay (n=5 biologically independent

mice). The tumor growth curve (B), two-way ANOVA; excised tumor weight (C), two-tailed unpaired *t* test. The tumor was subjected to Ki-67 staining (D, E), two-tailed unpaired *t* test. (F) ZDHHC20 IHC staining of tumor tissues in KPC mice infected with AAV-shControl or AAV-shZDHHC20. (G) Western blot analysis for the expression of ZDHHC20 in BxPC-3 and CAPAN-1 cells infected with lentivirus vectors expressing control or ZDHHC20, similar results were obtained in three independent experiments. (H, I) BxPC-3 (H) and CAPAN-1 (I) cells infected with lentivirus vectors expressing control or ZDHHC20 treated with DMSO or 2-BP were harvested for CCK-8 assay, n=3 biologically independent experiments, two-way ANOVA. (J, K) BxPC-3 (J) and CAPAN-1 (K) cells infected with lentivirus vectors expressing ZDHHC20 WT/C156S/F174A were harvested for CCK-8 assay, n=3 biologically independent experiments, two-way ANOVA. (L-N) PANC-1 cells infected with lentivirus vectors shControl, shZDHHC20 or shSTAT3 were injected subcutaneously into the nude mice for xenografts assay, n=6 biologically independent mice (L); Tumors were harvested, photographed, and weighed at day 22, one-way ANOVA (M); Tumor volumes were measured every 3 days, two-way ANOVA (N). (O-Q) PANC-1 cells infected with lentivirus vectors shControl, shZDHHC20 or BP-1-102 were injected subcutaneously into the nude mice for xenografts assay, n=6 biologically independent mice (O); Tumors were harvested, photographed, and weighed at day 22, one-way ANOVA (P); Tumor volumes were measured every 3 days, two-way ANOVA(Q). The box plots in (C, M, P) are defined in terms of minima, maxima, centre, bounds of box and whiskers and percentile. Statistical data presented in results (A, B, E, H-K, N, Q) show mean values ± SD. Source data are provided as a Source Data file.

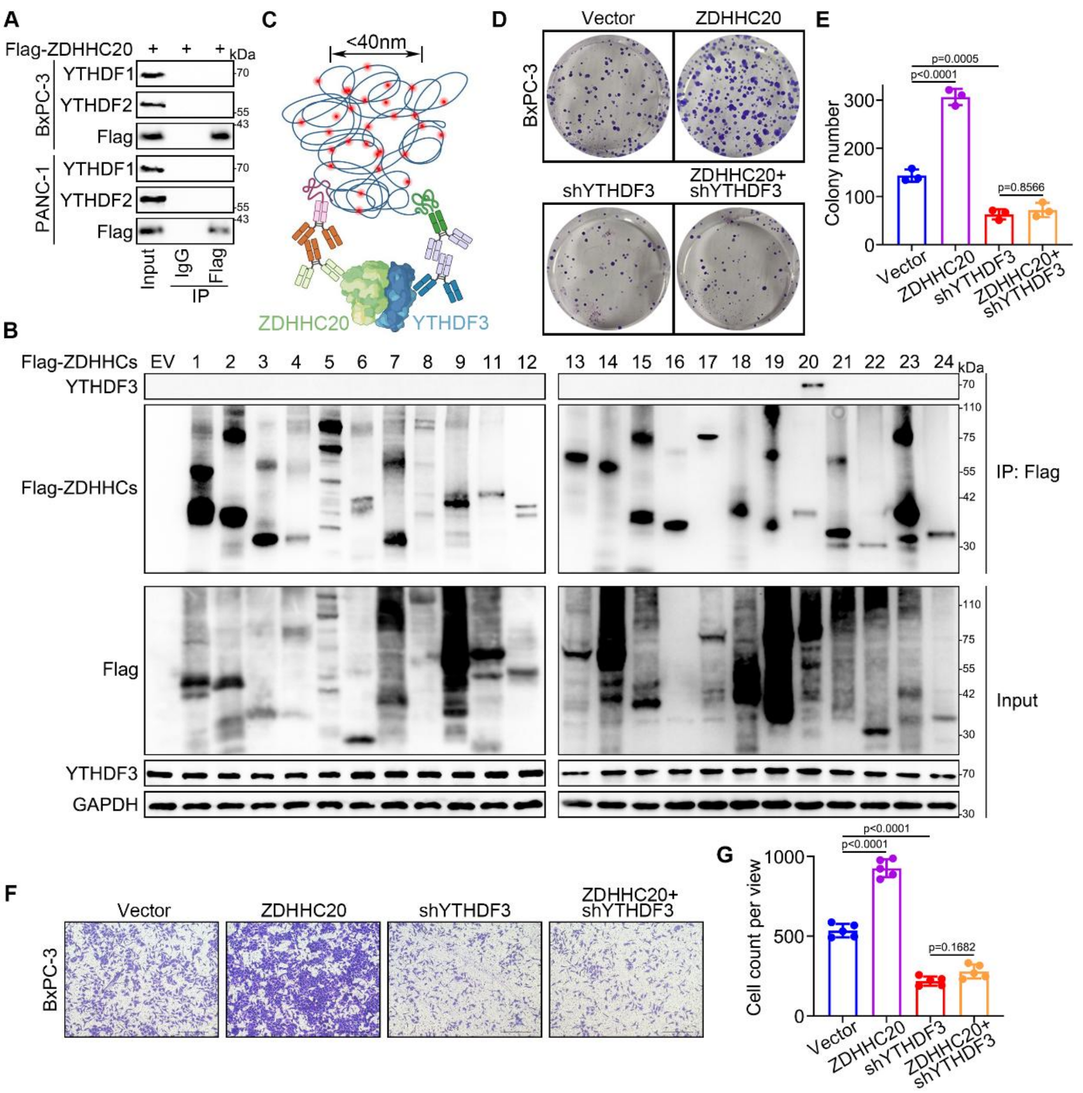

Figure S6. YTHDF3 mediates the oncogenic capacity of ZDHHC20 in pancreatic cancer

(A) Western blot analysis of YTHDF1, YTHDF2 and Flag-ZDHHC20 proteins reciprocally immunoprecipitated by anti- Flag and in PANC-1 and BxPC-3 cells. (B) Western blot analysis of all Flag-ZDHHCs and YTHDF3 proteins reciprocally immunoprecipitated by anti- Flag in PANC-1 cells. (C) Schematic of the Proximity Ligation Assay (PLA) used for the detection of transient interaction of endogenous ZDHHC20 with YTHDF3 in the cytoplasm of PANC-1 cells (Created with BioRender.com released under a CC-BY-NC-ND 4.0 International license). (D-G) BxPC-3 cells infected with lentivirus were harvested for for colony formation assay, n=3 biologically independent experiments (D, E); Transwell invasion assay, n=3 biologically independent experiments, scale bars: 100 μm (F, G); Each bar represents the mean ± SD, one-way ANOVA. Data are presented as mean ± SD. Similar results for (A, B) panels were obtained in three independent experiments. Source data are provided as a Source Data file.

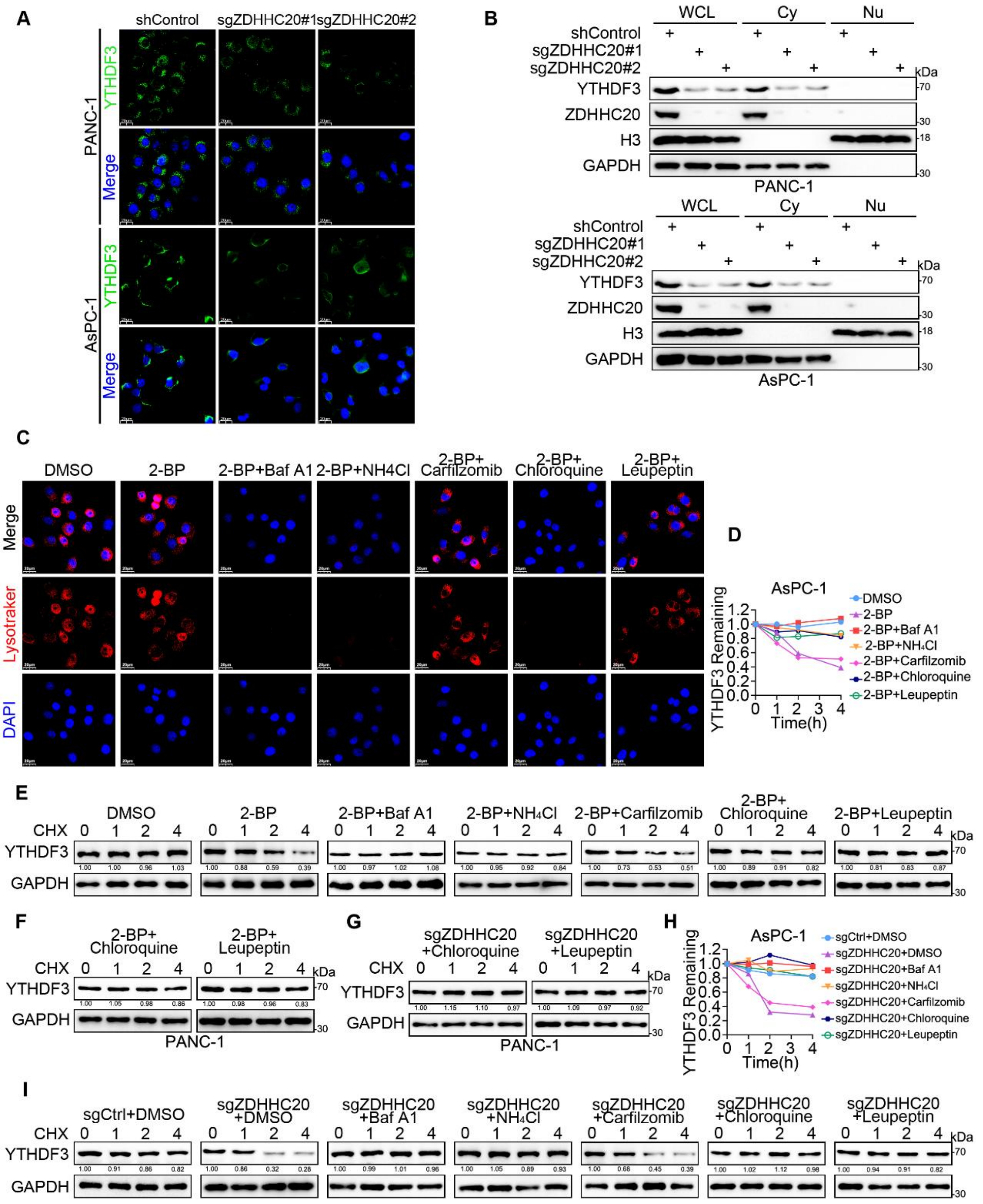

Figure S7. ZDHHC20 suppresses the lysosome-dependent degradation of YTHDF3

(A) Immunofluorescence confocal microscopy showed the localization of YTHDF3 in PANC-1 and BxPC-3 cells with or without ZDHHC20 knockout. Scale bar: 5 μm. (B) Nucleus/cytoplasmic YTHDF3 quantification of PANC-1 and AsPC-1 cells with or without ZDHHC20 knockout. (C) Representative images of lysotracker and DAPI immunofluorescence staining in PANC-1 cells treated with or without 2-BP in the presence of lysosomal inhibitors (bafilomycin A1, NH_4Cl, chloroquine and leupeptin) and proteasomal inhibitor Carfilzomib. Scale bar: 5 μm. (D, E) The degradation of

YTHDF3 in AsPC-1 cells treated with or without 2-BP was evaluated by CHX-chase assay in the presence of lysosomal inhibitors (bafilomycin A1, NH_4Cl, chloroquine and leupeptin) and proteasomal inhibitor Carfilzomib. Quantification of the intensity determined by the relative level of YTHDF3 remaining. (F) The degradation of YTHDF3 in PANC-1 cells treated with or without 2-BP was evaluated by CHX-chase assay in the presence of lysosomal inhibitors (chloroquine and leupeptin). (G) The degradation of YTHDF3 in PANC-1 cells treated with or without ZDHHC20 knockout was evaluated by CHX-chase assay in the presence of lysosomal inhibitors (chloroquine and leupeptin). (H, I) The degradation of YTHDF3 in AsPC-1 cells treated with or without ZDHHC20 knockout was evaluated by CHX-chase assay in the presence of lysosomal inhibitors (bafilomycin A1, NH_4Cl, chloroquine and leupeptin) and proteasomal inhibitor Carfilzomib. Quantification of the intensity determined by the relative level of YTHDF3 remaining. Similar results for (A-I) panels were obtained in three independent experiments. Source data are provided as a Source Data file.

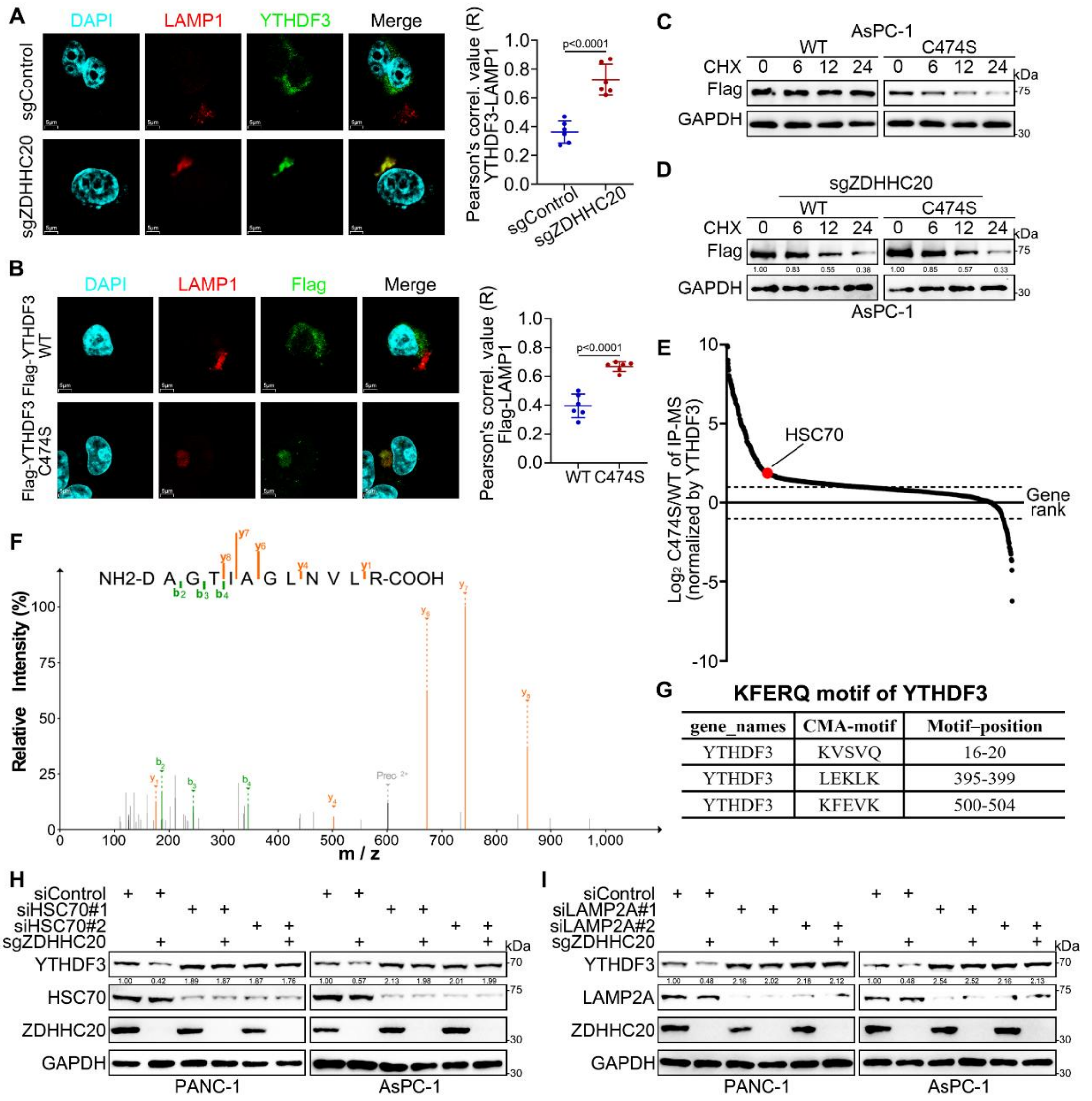

gene_names	CMA-motif	Motif–position
YTHDF3	KVSVQ	16-20
YTHDF3	LEKLK	395-399
YTHDF3	KFEVK	500-504

Figure S8. ZDHHC20 suppresses the chaperone-mediated autophagy of YTHDF3 via palmitoylation on Cys474

(A) Representative images of YTHDF3, LAMP1 and DAPI immunofluorescence staining in PANC-1 cells with or without ZDHHC20 knockout. Scale bar: 5 μm; n=6 biologically independent experiments, two-tailed unpaired *t* test. (B) Representative images of Flag, LAMP1 and DAPI immunofluorescence staining in PANC-1 cells infected with Flag-YTHDF3 WT/C474S plasmids. Scale bar: 5 μm; n=6 biologically independent experiments, two-tailed unpaired *t* test. (C) The degradation of Flag-YTHDF3 WT/C474S in AsPC-1 cells infected with Flag-YTHDF3 WT/C474S plasmids was evaluated by CHX-chase assay. (D) The degradation of Flag-YTHDF3 WT/C474S in ZDHHC20-KO AsPC-1 cells infected with Flag-YTHDF3 WT/C474S plasmids was evaluated by CHX-chase assay. (E) Mass spectrometry analysis of Flag-YTHDF3 WT/C474S immunoprecipitates in PANC-1 cell. The difference interaction proteins were normalized by bait protein YTHDF3 (F) Mass spectrometry

analysis of a peptide derived from Flag-YTHDF3 WT/C474S immunoprecipitates to show the HSC70 interaction between Flag-YTHDF3 WT/C474S. (G) The presence of three canonical KFERQ-like motifs in human YTHDF3. (H) Western blot analysis of PANC-1 and AsPC-1 cells infected with HSC70 siRNAs after endogenous ZDHHC20 knockout. (I) Western blot analysis of PANC-1 and AsPC-1 cells infected with LAMP2A siRNAs after endogenous ZDHHC20 knockout. Statistical data presented in results (A, B) show mean values ± SD. Similar results for (C, D, H, I) panels were obtained in three independent experiments. Source data are provided as a Source Data file.

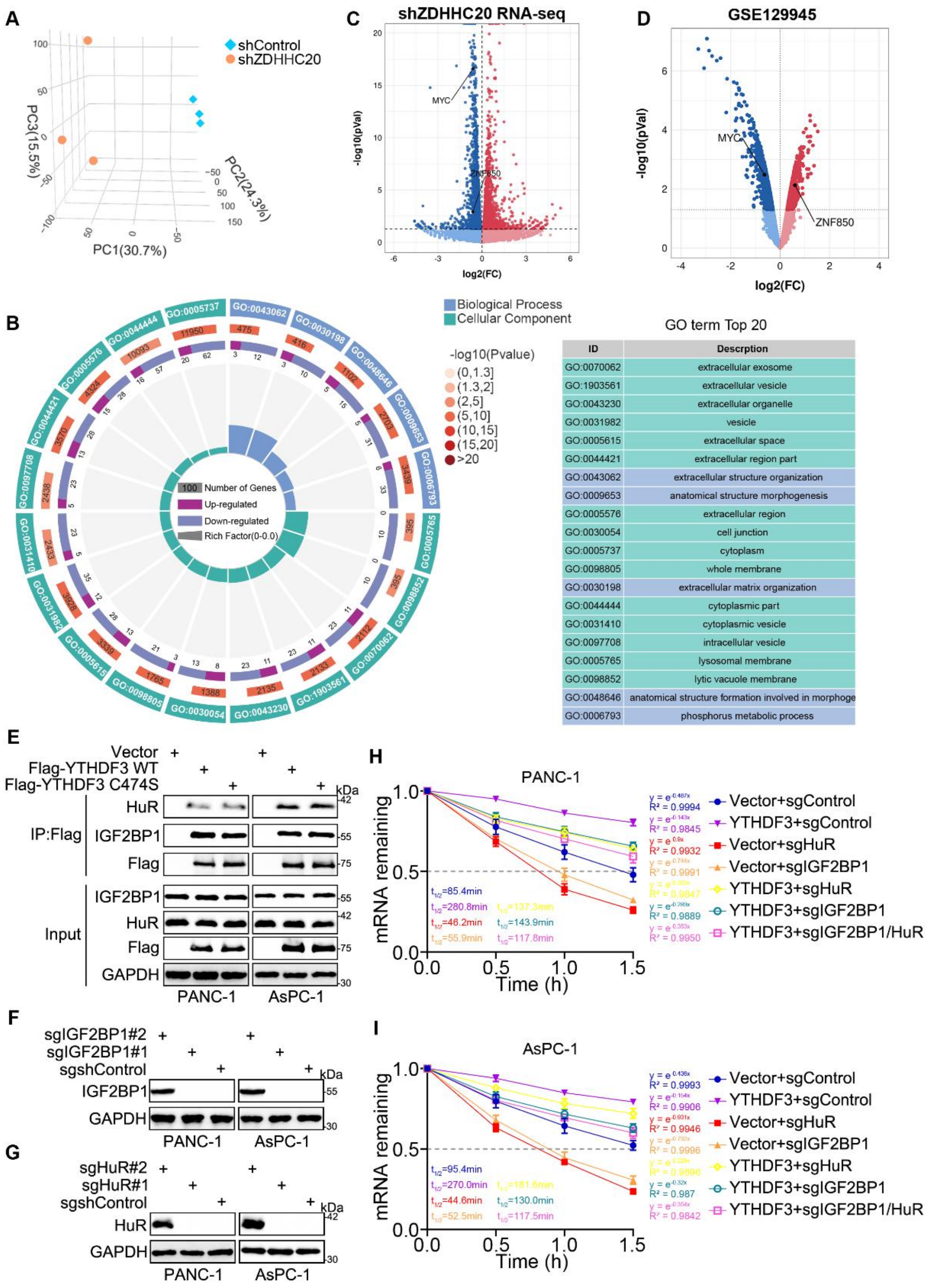

Figure S9. YTHDF3 stabilizes MYC mRNA in an m6A-dependent manner

(A) Principal component analysis (PCA) revealed the difference between the shZDHHC20 and control groups of the RNA-seq. (B) Gene Ontology (GO) enrichment analysis also suggested that ZDHHC20 regulated protein trafficking in lysosomes, vesicles, and the extracellular space. (C) Volcano plot analysis showed differential mRNA expression of the shZDHHC20 RNA-seq in PANC-1 cells. (D) Volcano plot analysis showed differential mRNA expression of the shYTHDF3

RNA-seq in GSE129945. (E) Western blot analysis of ectopically expressed Flag-YTHDF3 WT/C474S, IGF2BP1 and HuR reciprocally immunoprecipitated by anti-Flag in PANC-1 and AsPC-1 cells. (F) Western blot analysis for the expression of IGF2BP1 in PANC-1 and AsPC-1 cells with IGF2BP1 knockout. (G) Western blot analysis for the expression of HuR in PANC-1 and AsPC-1 cells with HuR knockout. (H, I) The half-life of MYC mRNA in PANC-1 (H) and AsPC-1 (I) cells infected with IGF2BP1/HuR knockout or YTHDF3 overexpression was evaluated by actinomycin D treatment for different time, the half-life of mRNA was estimated as equation in Method, nonlinear regression analysis. Each bar represents the mean ± SD of three independent experiments. Similar results for (E-G) panels were obtained in three independent experiments. Source data are provided as a Source Data file.

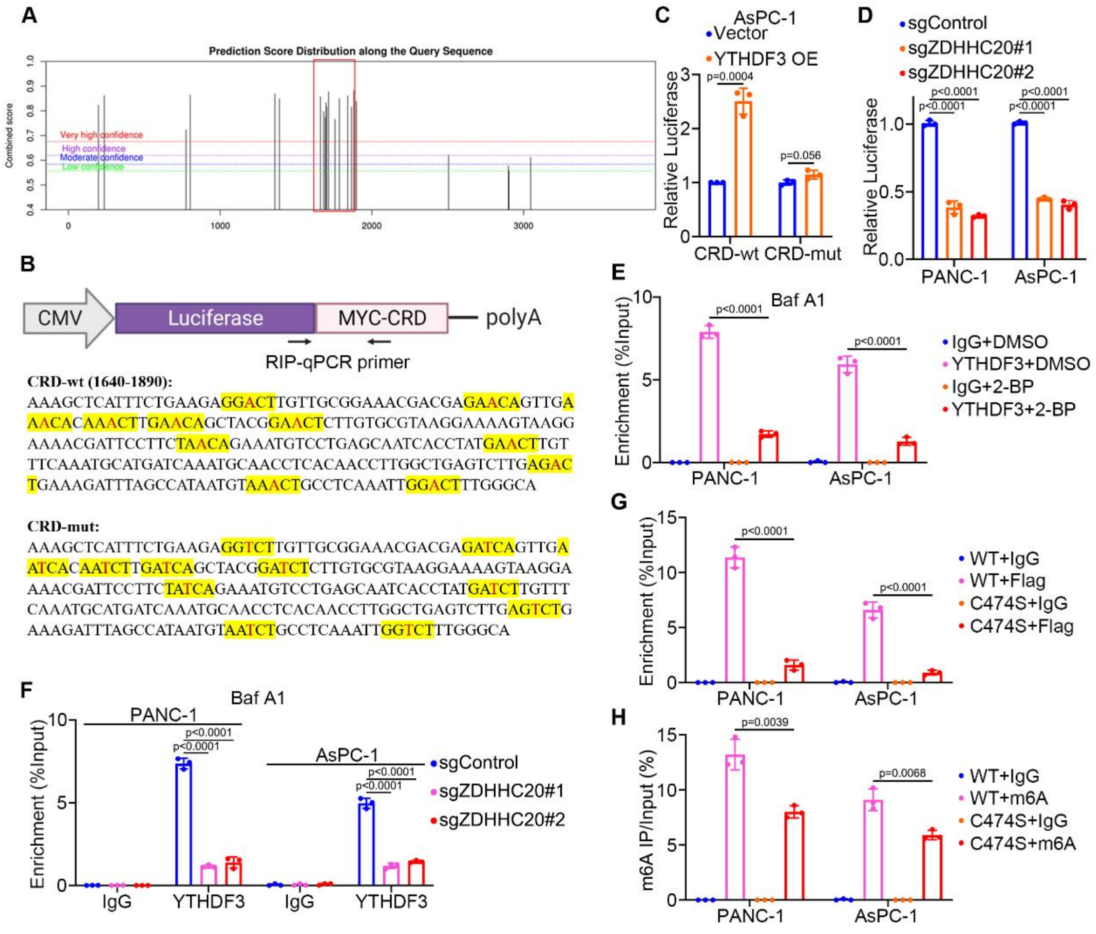

Figure S10. YTHDF3 recognizes m6A modification in the MYC CRD region

(A) Prediction of N6-methyladenosine (m6A) sites of MYC based on sequence-derived features by sequence-based RNA adenosine methylation site predictor (SRAMP) web server. The red frame indicated the MYC CRD domain. (B) Schematic diagram of the CRD-wt and CRD-mut. (C) Relative firefly luciferase (Fluc) activity of wild-type (CRD-wt) or mutated (CRD-mut) CRD reporters in AsPC-1 cells with ectopically expressed YTHDF3, n=3 biologically independent experiments, two-tailed unpaired *t* test. (D) Relative firefly luciferase (Fluc) activity of wild-type CRD reporters in PANC-1 and AsPC-1 cells with ZDHHC20 knockout, n=3 biologically independent experiments, one-way ANOVA. (E) RIP-qPCR assay of MYC by using the IgG or YTHDF3 antibodies in PANC-1 and AsPC-1 cells treated with 2-BP in the presence of bafilomycin A1, n=3 biologically independent experiments, two-tailed unpaired *t* test. (F) RIP-qPCR assay of MYC by using the IgG or YTHDF3 antibodies in ZDHHC20-KO PANC-1 and AsPC-1 cells in the presence of bafilomycin A1, n=3 biologically independent experiments, one-way ANOVA. (G) RIP-qPCR assay of MYC by using the IgG or Flag antibodies in PANC-1 and AsPC-1 cells infected with Flag-YTHDF3 WT/C474S plasmids, n=3 biologically independent experiments, two-tailed unpaired *t* test. (H) MeRIP-qPCR assay of MYC by using the

IgG or m6A antibodies in PANC-1 and AsPC-1 cells infected with Flag-YTHDF3 WT/C474S plasmids, n=3 biologically independent experiments, two-tailed unpaired *t* test. Statistical data presented in results (C-H) show mean values ± SD. Source data are provided as a Source Data file.

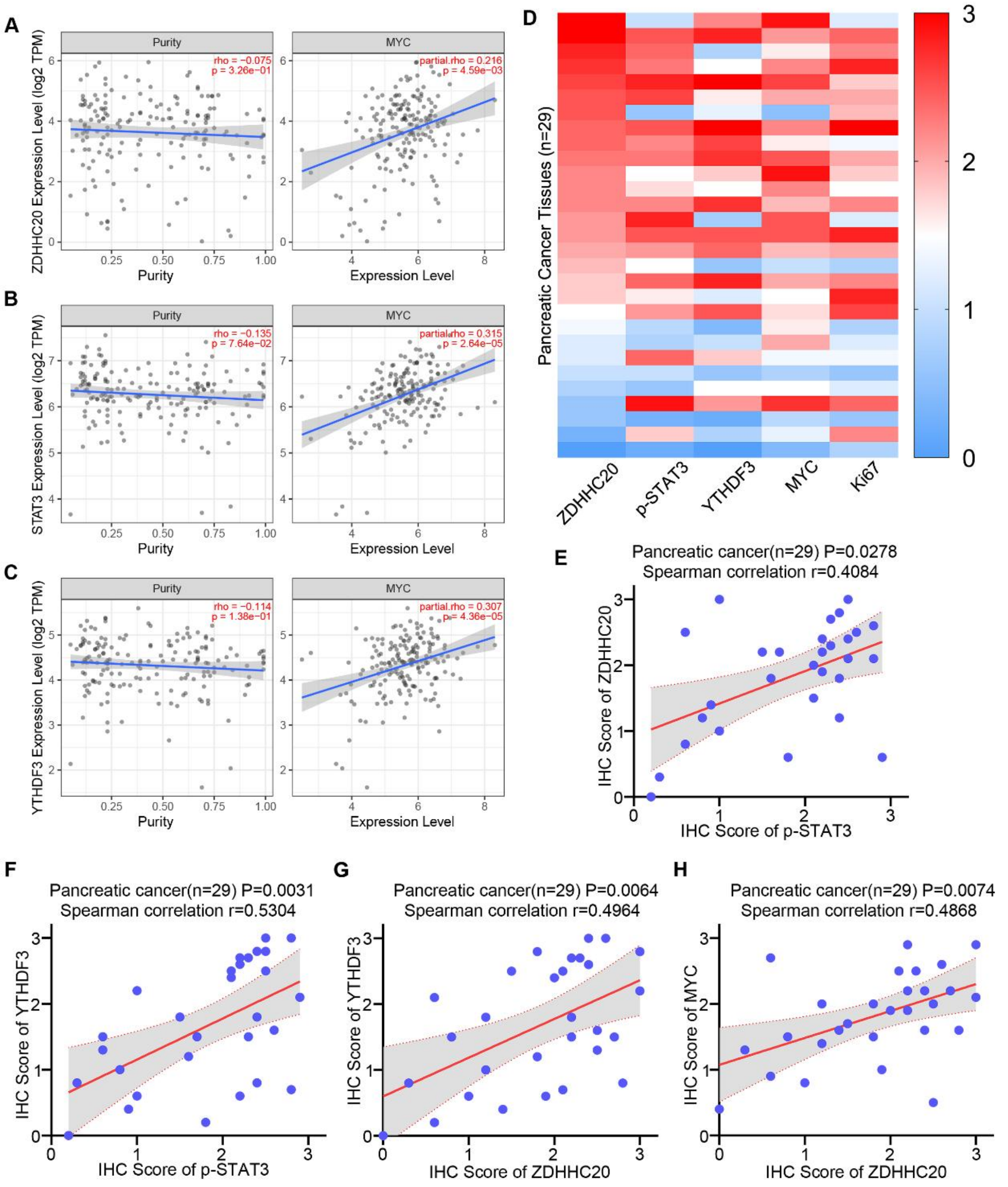

Figure S11. A robust positive association between MYC and those of ZDHHC20, STAT3 and YTHDF3

(A) TIMER2.0 showed purity-adjusted correlation between MYC and ZDHHC20 in PAAD data set. (B) TIMER2.0 showed purity-adjusted correlation between MYC and STAT3 in PAAD data set. (C) TIMER2.0 showed purity-adjusted correlation between MYC and YTHDF3 in PAAD data set. (D) The heatmap revealed IHC staining score of ZDHHC20, p-STAT3, YTHDF3, MYC and Ki67 on TMA containing a cohort of pancreatic cancer samples (n=29). (E) The correlation analysis of IHC staining index of p-STAT3 and ZDHHC20 on TMA containing a cohort of pancreatic cancer samples (n=29). (F) The correlation analysis of IHC staining index of p-STAT3 and YTHDF3 on TMA containing a cohort of pancreatic cancer samples (n=29). (G) The correlation

analysis of IHC staining index of ZDHHC20 and YTHDF3 on TMA containing a cohort of pancreatic cancer samples (n=29). (H) The correlation analysis of IHC staining index of ZDHHC20 and MYC on TMA containing a cohort of pancreatic cancer samples (n=29). Source data are provided as a Source Data file.

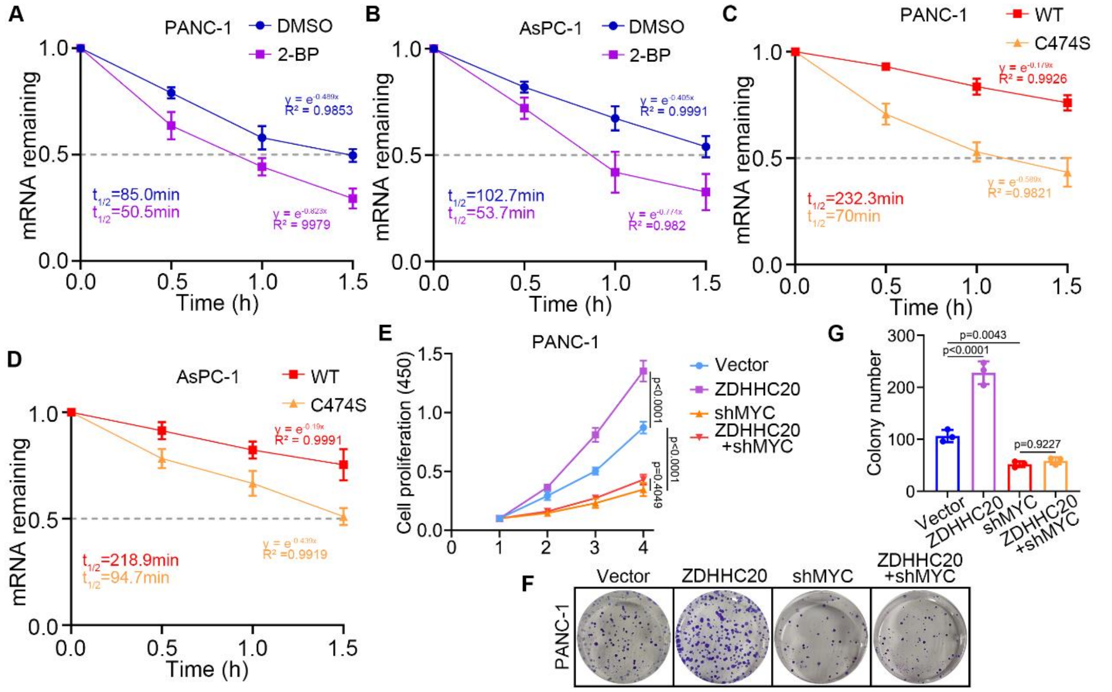

Figure S12. ZDHHC20-mediated palmitoylation of YTHDF3-Cys474 stabilizes MYC mRNA

(A, B) The half-life of MYC mRNA in PANC-1 (A) and AsPC-1 (B) cells treated with DMSO/2-BP was evaluated by actinomycin D treatment for different time. Each bar represents the mean ± SD of three independent experiments, the half-life of mRNA was estimated as equation in Method, nonlinear regression analysis. (C, D) The half-life of MYC mRNA in PANC-1 (C) and AsPC-1 (D) cells infected with Flag-YTHDF3 WT/C474S plasmids was evaluated by actinomycin D treatment for different time, the half-life of mRNA was estimated as equation in Method, nonlinear regression analysis. Each bar represents the mean ± SD of three independent experiments. (E-G) PANC-1 cells infected with lentivirus were harvested for CCK-8 assay, n=3 biologically independent experiments, two-way ANOVA (E); Colony formation assay, n=3 biologically independent experiments, one-way ANOVA (F, G). Data are presented as mean ± SD. Source data are provided as a Source Data file.

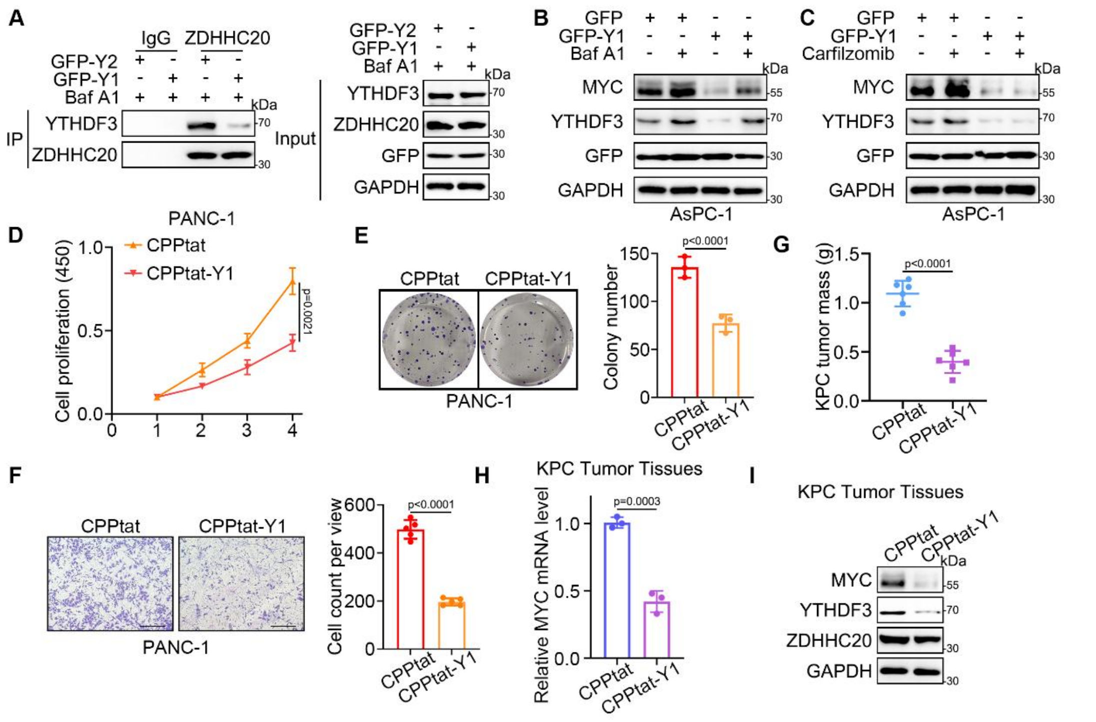

Figure S13. Therapeutic blockade of the ZDHHC20-YTHDF3 interaction inhibits pancreatic cancer progression

(A) Under Barf A1 treatment, western blot analysis of endogenous ZDHHC20 and YTHDF3 proteins immunoprecipitated by anti-ZDHHC20 in PANC-1 cells infected with GFP-Y1 and GFP-Y2. (B) Western blot analysis for the expression of GFP, YTHDF3 and MYC in AsPC-1 cells infected with GFP-Vector or GFP-Y1 plasmids, with or without Baf A1 treatment. (C) Western blot analysis for the expression of GFP, YTHDF3 and MYC in AsPC-1 cells infected with GFP-Vector or GFP-Y1 plasmids, with or without Carfilzomib treatment. (D-F) PANC-1 cells treated with CPPtat/ CPPtat-Y1 were harvested for CCK-8 assay, n=3 biologically independent experiments, two-way ANOVA (D); Colony formation assay, n=3 biologically independent experiments, two-tailed unpaired *t* test (E); Transwell invasion assay, scale bars: 100 μm. n=5 biologically independent experiments, two-tailed unpaired *t* test (F). (G) The comparison of pancreas mass in KPC mice after CPPtat/ CPPtat-Y1 treatment. n =6 biologically independent mice, two-tailed unpaired *t* test. (H) RT-qPCR analysis for the mRNA expression level of MYC in pancreatic tumor tissues from KPC mice treated with CPPtat/ CPPtat-Y1, n=3 biologically independent experiments, two-tailed unpaired *t* test. (I) Western blot analysis for the expression of MYC, YTHDF3 and ZDHHC20 in pancreatic tumor tissues from KPC mice treated with CPPtat/ CPPtat-Y1. Statistical data presented in results (D-H) show mean values ± SD. Similar results for (A, C, I) panels were obtained in three independent experiments. Source data are provided as a Source Data file.

Table S1. Sequence of primers and gene specific shRNAs, sgRNAs & siRNAs

**shRNAs**	**Sequence**
shZDHHC20#1	CCGGGCAAAGAATCACATGCCACTTCTCGAGAAGTGGCATGTGATTCTTTGCTTTTT
shZDHHC20#2	CCGGGAACAAGCTTCTGTTACAACTCGAGTTGTAACAGAAGCTTGTTCTTTTT
shYTHDF3#1	CCGGGTCAGTGCTTCACCTTCTACTCGAGTAGAAGGTGAAGCACTGACTTTTT
shYTHDF3#2	CCGGCATACATCGTTCCATTAAACTCGAGTTTAATGGAACGATGTATGTTTTT
shMYC#1	CCGGGAGAATGTCAAGAGGCGAACTCGAGTTCGCCTCTTGACATTCTCTTTTT
shMYC#2	CCGGGTTGCGGAAACGACGAGAACTCGAGTTCTCGTCGTTTCCGCAACTTTTT
sh-mZDHHC20	CCGGTCAGTGTGCTCTCACTATTTACTCGAGTAAATAGTGAGAGCACACTGATTTTT
**sgRNAs**	**Sequence**
sgZDHHC20#1	CGTATGAAAACGTGGGTGCGCGG
sgZDHHC20#2	GTAGGACCAGACGACCACGA
sgYTHDF3#1	CTAAGCGAATATGCCGTAATTGG
sgYTHDF3#2	TGGGTAGCTCCTCGTAACAGGGG
**siRNAs**	**Target Sequence**
siSTAT3#1	GCACAATCTACGAAGAATCAA
siSTAT3#2	GCAACAGATTGCCTGCATTGG
siHSC70#1	CGUCUGAUUGGACGCAGAUUUTT
siHSC70#2	CCAAGACUUCUUCAAUGGAAATT
siLAMP2A#1	GAAGUGAACAUCAGCAUGUAUTT
siLAMP2A#2	GCCAUCAGAAUUCCAUUGAAUTT

Table S2. The sequences of primers, oligos and m6A-oligos used in this study.

Gene	Usage	Forward primer (5′ - 3′)	Reverse primer (5′ - 3′)
ZDHHC20	RT-qPCR	CGCACCCACGTTTTC ATACG	TCTGGCATACTCATTC TGGTTTG
STAT3	RT-qPCR	TCTGGCATACTCATTC TGGTTTG	TCCATCGCTGACAAA AGCCC
YTHDF3	RT-qPCR	GCAGAGGAAACAGG CGAAGAA	GGATCTGACATTGGT GGATAGCTG
MYC	RT-qPCR	ACACTAACATCCCAC GCTCTG	AAACCGCATCCTTGT CCTGTG
GAPDH	RT-qPCR	CAATGACCCCTTCAT TGACC	TTGATTTTGGAGGGA TCTCG
ZDHHC20	ChIP-qPC R	CAGACGACCACGAA GGTGATG	TCGGACTTTTGCTCC CACAA
MYC-CRD	RIP-qPCR	TGAAGAGGACTTGTT GCGGA	CTCAGCCAAGGTTGT GAGGTT
Luc-CRD	RIP-qPCR	GCGGAAAGATCGCCG TGTAAA	CAAGACTCAGCCAA GGTTGTGA
MYC-CRD A	RNA pulldown	Biotin-AGUUGAAACACAAACUUGAACAGCUA CGGAACUCUU	
MYC-CRD m6A	RNA pulldown	Biotin-AGUUGAAA*(m6A)CACAAA*(m6A)CUU GAA*(m6A)CAGCUACGGAA*(m6A)CUCUU	
