# Supplementary material for: ZDHHC20-mediated S-palmitoylation of YTHDF3 stabilizes MYC mRNA to promote pancreatic cancer progression
Source: Nat Commun. 2024 May 31;15:4642. doi: 10.1038/s41467-024-49105-3 (PMC11143236; doi:10.1038/s41467-024-49105-3)
Supplement: Supplementary file 3 — Description of Additional Supplementary Files [file 41467_2024_49105_MOESM3_ESM.pdf]

**The Supplemental files includes:**

- Supplementary Data1.

  Description: Supplementary Table S3. Information of Reagent or Resource.

- Supplementary Data2.

  Description: Supplementary Table S4. Mass spectrometry analysis of a peptide derived from Flag-ZDHHC20-immunoprecipitates to show the interaction proteins of ZDHHC20.

- Supplementary Data3.

  Description: Mass spectrometry analysis of a peptide derived from Flag-YTHDF3 WT/C474S-immunoprecipitates to show the difference interactions between YTHDF3 WT and YTHDF3 C474S.
