# Supplementary material for: ZDHHC20-mediated S-palmitoylation of YTHDF3 stabilizes MYC mRNA to promote pancreatic cancer progression
Source: Nat Commun. 2024 May 31;15:4642. doi: 10.1038/s41467-024-49105-3 (PMC11143236; doi:10.1038/s41467-024-49105-3)
Supplement: Supplementary file 7 — Reporting Summary [file 41467_2024_49105_MOESM7_ESM.pdf]

nature portfolio

Corresponding author(s): Xin Jin, Ke Jiang and Lu Tang

Last updated by author(s): Mar 8, 2024

Reporting Summary

Nature Portfolio wishes to improve the reproducibility of the work that we publish. This form provides structure for consistency and transparency in reporting. For further information on Nature Portfolio policies, see our Editorial Policies and the Editorial Policy Checklist.

Statistics

For all statistical analyses, confirm that the following items are present in the figure legend, table legend, main text, or Methods section.

n/a	Confirmed	
☐	☒	The exact sample size (*n*) for each experimental group/condition, given as a discrete number and unit of measurement
☐	☒	A statement on whether measurements were taken from distinct samples or whether the same sample was measured repeatedly
☐	☒	The statistical test(s) used AND whether they are one- or two-sided<br>*Only common tests should be described solely by name; describe more complex techniques in the Methods section.*
☒	☐	A description of all covariates tested
☐	☒	A description of any assumptions or corrections, such as tests of normality and adjustment for multiple comparisons
☐	☒	A full description of the statistical parameters including central tendency (e.g. means) or other basic estimates (e.g. regression coefficient) AND variation (e.g. standard deviation) or associated estimates of uncertainty (e.g. confidence intervals)
☐	☒	For null hypothesis testing, the test statistic (e.g. *F*, *t*, *r*) with confidence intervals, effect sizes, degrees of freedom and *P* value noted<br>*Give P values as exact values whenever suitable.*
☒	☐	For Bayesian analysis, information on the choice of priors and Markov chain Monte Carlo settings
☒	☐	For hierarchical and complex designs, identification of the appropriate level for tests and full reporting of outcomes
☒	☐	Estimates of effect sizes (e.g. Cohen's *d*, Pearson's *r*), indicating how they were calculated

Our web collection on statistics for biologists contains articles on many of the points above.

Software and code

Policy information about availability of computer code

Data collection	Tumor burdens were monitored and quantified by Bioluminescence imaging (BLI) on Lago X Imaging System (Spectral Instruments Imaging, USA). Transcriptome sequencing was performed by based on the Illumina sequencing platform. The mass spectrometry was performed using Thermo's Q Exactive Plus LMS system.
Data analysis	Data analysis and statistical tests were done using Prism (GraphPad Prism 9) or Excell (Microsoft). Confocal immunofluorescence images were analyzed with ImageJ (ImageJ). The intensity of immunoblotting bands was quantified by the Image Lab software.Gene set enrichment analyses were performed on differentially expressed genes using clusterProfiler package

For manuscripts utilizing custom algorithms or software that are central to the research but not yet described in published literature, software must be made available to editors and reviewers. We strongly encourage code deposition in a community repository (e.g. GitHub). See the Nature Portfolio guidelines for submitting code & software for further information.

Data

Policy information about availability of data

All manuscripts must include a data availability statement. This statement should provide the following information, where applicable:

- Accession codes, unique identifiers, or web links for publicly available datasets
- A description of any restrictions on data availability
- For clinical datasets or third party data, please ensure that the statement adheres to our policy

GSE16515（https://www.ncbi.nlm.nih.gov/geo/query/acc.cgi?acc=GSE16515）
GSE130173 (https://www.ncbi.nlm.nih.gov/geo/query/acc.cgi?acc=GSE130173)
The RNA-seq data generated in this study have been deposited in the NCBI Gene Expression Omnibus (GEO) under accession code GSE235516(https://www.ncbi.nlm.nih.gov/geo/query/acc.cgi?acc=GSE235516). All mass spectrometry raw data generated in this study have been deposited to the ProteomeXchange via the PRIDE partner repository with the dataset identifiers PXD046541(https://proteomecentral.proteomexchange.org/cgi/GetDataset?ID=PXD046541) and PXD043292(https://proteomecentral.proteomexchange.org/cgi/GetDataset?ID=PXD043292). All data needed to evaluate the conclusions in the paper are present in the paper and/or the Supplementary Materials. Additional data related to this paper may be requested from the corresponding authors (Xin Jin, jinxinxy2@csu.edu.cn) on reasonable request. Source data are provided with this paper.

Research involving human participants, their data, or biological material

Policy information about studies with human participants or human data. See also policy information about sex, gender (identity/presentation), and sexual orientation and race, ethnicity and racism.

Reporting on sex and gender	Tissues samples were collected from 10 patients, including 4 women and 6 men. Informed consent from all participants has been obtained. Sex and gender were not considered in this study.
Reporting on race, ethnicity, or other socially relevant groupings	All the volunteers are Chinese. They were not categorized.
Population characteristics	See above.
Recruitment	All pancreatic cancer tissues and adjacent normal tissues (n=10) were recruited from patients with who underwent a pancreatic ductal adenocarcinoma surgical resection. All individuals provided informed consent.
Ethics oversight	All experiments were approved by the Union Hospital of Tongji Medical College; Huazhong University of Science and Technology(permit number [2020]IEC-J(030))

Note that full information on the approval of the study protocol must also be provided in the manuscript.

Field-specific reporting

Please select the one below that is the best fit for your research. If you are not sure, read the appropriate sections before making your selection.

☒ Life sciences ☐ Behavioural & social sciences ☐ Ecological, evolutionary & environmental sciences

For a reference copy of the document with all sections, see nature.com/documents/nr-reporting-summary-flat.pdf

Life sciences study design

All studies must disclose on these points even when the disclosure is negative.

Sample size	Sample sizes for each experiment are provided in figure legends. For in vitro experiments, three or five biological replicates were achieved. Such sample sizes are typical for the in vitro experiments and sufficient for a statistical analysis. For in vivo assay, n=6 or n=8 mice were used. The sample sizes are determined empirically, and are similar in size to most existing studies in the field
Data exclusions	No data were excluded from analysis.
Replication	Each in vitro experiment was replicated at least triple successfully. At least five mice and human samples were used in this study and similar results were obtained in independent experiments.
Randomization	The samples for each experiment were randomized to be examined (No specific methods were used).
Blinding	No blinding was performed due to none of the analyses reported involved procedures that could be influenced by investigator bias.

Reporting for specific materials, systems and methods

We require information from authors about some types of materials, experimental systems and methods used in many studies. Here, indicate whether each material, system or method listed is relevant to your study. If you are not sure if a list item applies to your research, read the appropriate section before selecting a response.

Materials & experimental systems

n/a	Involved in the study
[ ]	[x] Antibodies
[ ]	[x] Eukaryotic cell lines
[x]	[ ] Palaeontology and archaeology
[ ]	[x] Animals and other organisms
[x]	[ ] Clinical data
[x]	[ ] Dual use research of concern
[x]	[ ] Plants

Methods

n/a	Involved in the study
[x]	[ ] ChIP-seq
[x]	[ ] Flow cytometry
[x]	[ ] MRI-based neuroimaging

Antibodies

Antibodies used	Rabbit polyclonal anti-ZDHHC20 Abmart Cat#TD4335, working dilution 1:500<br>Rabbit polyclonal anti-YTHDF3 Proteintech Cat#25537-1-AP, working dilution 1:1000<br>Mouse monoclonal anti-ZDHHC20 Santa Cruz Biotechnology Cat#sc-518217, working dilution 1:500<br>Mouse monoclonal anti-YTHDF3 Santa Cruz Biotechnology Cat#sc-377119, working dilution 1:1000<br>Rabbit polyclonal anti-MYC Proteintech Cat#10828-1-AP, working dilution 1:1000<br>Rabbit polyclonal anti-STAT3 Proteintech Cat#10253-2-AP, working dilution 1:1000<br>Rabbit polyclonal anti- Phospho-STAT3 (Tyr705) Cell Signaling Technology Cat#9131, working dilution 1:1000<br>Rabbit polyclonal anti-KRAS Proteintech Cat#12063-1-AP, working dilution 1:1000<br>Rabbit polyclonal anti-GAPDH Proteintech Cat# 10494-1-AP, working dilution 1:5000<br>Rabbit monoclonal anti- Flag tag Cell Signaling Technology Cat# 14793, working dilution 1:2000<br>Mouse monoclonal anti- Flag tag Proteintech Cat# 66008-4-Ig, working dilution 1:2000<br>Rabbit monoclonal anti- HA tag Abcam Cat# ab236632, working dilution 1:1000<br>Rabbit polyclonal anti-GST tag Antibody Proteintech Cat#10000-0-AP, working dilution 1:2000<br>Rabbit polyclonal anti-Streptavidin-HRP Proteintech Cat# SA00001-0, working dilution 1:1000<br>Rabbit polyclonal anti-GFP tag Proteintech Cat# 50430-2-AP, working dilution 1:2000<br>Rabbit polyclonal anti-LAMP2 Proteintech Cat# 27823-1-AP, working dilution 1:1000<br>Rabbit polyclonal anti- Histone-H3 Sigma-Aldrich Cat#H0164, working dilution 1:5000<br>Rabbit monoclonal anti- YTHDF1 Cell Signaling Technology Cat#57530, working dilution 1:1000<br>Rabbit polyclonal anti-YTHDF2 Proteintech Cat#24744-1-AP, working dilution 1:1000<br>Rabbit polyclonal anti-LAMP-2A Thermofisher Cat#51-2200, working dilution 1:1000<br>Rabbit polyclonal anti-HSC70 Proteintech Cat#10654-1-AP, working dilution 1:1000<br>Rabbit monoclonal anti- LAMP1 Cell Signaling Technology Cat# 9091, working dilution 1:1000<br>Rabbit IgG Beyotime A7016<br>Mouse Anti-Rabbit IgG (Light-Chain Specific) mAb Cell Signaling Technology Cat #93702, working dilution 1:5000
Validation	All antibodies were validated by the suppliers for the applications in their data sheets or manufacturers' websites that we used in this study, and were checked in our lab by comparing to vendors' results.

Eukaryotic cell lines

Policy information about cell lines and Sex and Gender in Research

Cell line source(s)	The pancreatic cancer cell lines (SW1990, AsPC-1, MIAPaCa-2, CFPAC1, PANC-1, CAPAN-1 and BxPC-3), normal human pancreatic duct epithelial cells (HPDE6-C7) and HEK293T were purchased from the ATCC (USA), National Collection of Authenticated Cell Cultures (Shanghai, China) and Procell Life Science&Technology Co., Ltd (Wuhan, China).
Authentication	All cell lines used in this study were authenticated with STR profiling
Mycoplasma contamination	All cell lines tested negative for mycoplasma contamination.
Commonly misidentified lines<br>(See ICLAC register)	No misidentified cell lines were used in this study

Animals and other research organisms

Policy information about studies involving animals; ARRIVE guidelines recommended for reporting animal research, and Sex and Gender in Research

Laboratory animals	The BALB/c-nu mice (4 weeks old, male) and M-NSG (NOD-Prkdcscid IL2rgem1/Smoc, male, 4 weeks old) mice were purchased from Vitalriver (Beijing, China) and housed under pathogen-free conditions for one week before the experiments. The KPC (LSL-KrasG12D/

	+; LSL-Trp53R172H/+; Pdx-1-Cre;8 weeks old) transgenic mice were purchased from MODEL ORGANISMS Inc. (Shanghai, China) and housed under pathogen-free conditions. Mice were housed in standard cages with an SPF environment with a 12-hour light/dark cycle at a room temperature of 22±2℃, humidity of 50±5%, with free access to water and a standard diet .
Wild animals	No wild animals were used.
Reporting on sex	All animals used in one experiment were consistent, and no sex-based analysis was performed.
Field-collected samples	No field collected samples were used in the study.
Ethics oversight	This study was approved by the institutional research ethics committee of Tongji Medical College, Huazhong University of Science and Technology, all animal experiments were performed in strict accordance with the recommendations in the guide for the care and Use of laboratory animals of Tongji Medical College. The licence was issued by the Animal Use and Care Committees at Tongji Medical College, Huazhong University of Science and Technology (IACUC Number 2728).

Note that full information on the approval of the study protocol must also be provided in the manuscript.
